# Self Perceptions as Predictors for Return to Work 2 Years After Rehabilitation in Orthopedic Trauma Inpatients

**DOI:** 10.1007/s10926-012-9369-x

**Published:** 2012-05-05

**Authors:** Maria Iakova, Pierluigi Ballabeni, Peter Erhart, Nikola Seichert, François Luthi, Olivier Dériaz

**Affiliations:** 1 Département de l’appareil locomoteur, Clinique Romande de Réadaptation SUVA Care, Avenue Grand-Champsec 90, 1951 Sion, Switzerland; 2 Research Department, Clinique Romande de Réadaptation SUVA Care, Sion, Switzerland; 3 Institut de recherche en réadaptation et Service de recherche médicale, Clinique Romande de Réadaptation SUVA Care, Sion, Switzerland; 4 Department of Vocational Rehabilitation, Rehaklinik Bellikon, Bellikon, Switzerland; 5 Département de l’appareil locomoteur, Centre Hospitalier Universitaire Vaudois, University of Lausanne, Lausanne, Switzerland; 6 Institute of Social and Preventive Medicine (IUMSP), Centre Hospitalier Universitaire Vaudois, University of Lausanne, Lausanne, Switzerland

**Keywords:** Return to work, Injury, Accident, Pain, PTSD, IES-R

## Abstract

*Purpose* This study aimed to identify self-perception variables which may predict return to work (RTW) in orthopedic trauma patients 2 years after rehabilitation. *Methods* A prospective cohort investigated 1,207 orthopedic trauma inpatients, hospitalised in rehabilitation, clinics at admission, discharge, and 2 years after discharge. Information on potential predictors was obtained from self administered questionnaires. Multiple logistic regression models were applied. *Results* In the final model, a higher likelihood of RTW was predicted by: better general health and lower pain at admission; health and pain improvements during hospitalisation; lower impact of event (IES-R) avoidance behaviour score; higher IES-R hyperarousal score, higher SF-36 mental score and low perceived severity of the injury. *Conclusion* RTW is not only predicted by perceived health, pain and severity of the accident at the beginning of a rehabilitation program, but also by the changes in pain and health perceptions observed during hospitalisation.

## Background

After traumatic injury, most patients will generally return to work within a few weeks. However, the majority of the costs will be caused by the few remaining workers with long term sick leaves [1]. For this reason, it is important for clinicians to identify, as early as possible, workers at high risk for disability in order to facilitate intervention strategies [1].

No consensus exists in the literature and various prognostic factors have been proposed (for a review see [2]). However, there is increasing evidence that the return to work may be predicted by some variables like education, gender, blue collar, injury severity, number of surgical procedures and self-efficacy (psychological factors) [2]. The earliest studies, based on biomedical variables failed to predict RTW [3]. This prompted the development of biopsychosocial models (BPS) [4, 5] which aims to analyze the multidimensional nature of the problem and several predictors have been proposed. Work-ability, which is linked to return to work, depends on factors related to the patient (physical, psychological, cognitive, and behavioural factors) but also to the environment (social, workplace factors and factors outside the workplace) [6]. Taking all these results together, it appears that psychosocial factors are of primary importance. For instance, subjective perception of pain, self-assessment of physical status [7], patient beliefs [8] and catastrophising [9, 10] as well as fear avoidance [11] are good predictors of disease chronicity and, consequently, RTW. Finally, post traumatic stress disorder (PTSD), which was initially studied in soldiers, is also present in patients with musculoskeletal trauma (for a review see [12]) and some studies have shown that PTSD is associated with return to work in patients with hand injury [13, 14] and burns [15] but these results were not reproduced by others in trauma patients [16]. This important issue remains to be investigated more thoroughly.

It can be postulated that the changes in prognostic factors, induced by a rehabilitation program (i.e. pain, psychological variables etc.), are good indicators of the patient’s response to the treatment and, consequently, may improve his/her outcome. This hypothesis is confirmed by few prospective studies in which changes in pain, perceived health/disability, related to the treatment, predicted return to work [17, 18]. However, this issue remains, to our knowledge, largely unexplored.

With the present prospective cohort study, we intended to investigate whether a number of baseline self-perception variables may predict return to work 2 years after orthopedic and vocational rehabilitation for orthopedic trauma. For this purpose, we analyzed data from patients recruited into a cohort called OUTCOME, started in two Swiss rehabilitation clinics with the aim of assessing quality of life and work status outcomes after rehabilitation [19].

## Methods

### Study Design

A prospective cohort study was conducted, in which self-administered questionnaires were used at admission into rehabilitation clinic, then at discharge and 2 years after discharge.

### Population

We included in this study patients with orthopedic trauma of the back and upper and lower limb, hospitalised in two Swiss rehabilitation clinics between 15 November 2003 and 31 December 2005. The clinics were the French speaking Clinique Romande de Réadaptation (CRR) at Sion, and the German speaking Rehaklinik Bellikon (RKB) at Bellikon. All patients hospitalised for a rehabilitation program after a traumatic injury were eligible for the OUTCOME study, if they had no severe traumatic brain injury (Glasgow coma scale ≤8), had no spinal cord injury, were capable of judgment, were not under legal custody and were not older than 60 years (considered as too old to have a reasonable chance to RTW). Most of our inpatients were blue collar workers and took part in a rehabilitation program after work, leisure or traffic accidents. Patients were sent to the rehabilitation hospitals when they presented persistent pain and functional limitations after an accident (median: 9 months after the accident). The aim of the therapeutic program is to take care of patients with an interdisciplinary approach (somatic, psychological and social) in order to reduce disabilities and improve chance of returning to work (usual or adapted to impairments).

Patients signed an informed consent form before entering the study. The protocol was approved by the ethical committee of the local medical associations.

### Variables

For the present analysis, baseline variables (predictors and confounders) were assessed by means of self-evaluation questionnaires filled in by the patients within 3 days after hospitalisation and 2 days before discharge. RTW status was assessed via a postal questionnaire sent 2 years after clinic discharge. The binary outcome was coded 1 if a subject had a job and 0 if a subject had no job.

The following variables were tested as potential predictors: (1) general health perceived at admission (visual analogue scale, VAS, scale range 0–100); (2) general health improvement during hospitalisation (VAS); (3) pain at admission (VAS, range 0–100); (4) pain decrease during hospitalisation (VAS); (5) anxiety score of Hospital Anxiety and Depression Scale (HADS) [20] at admission (range 0–21); (6) depression score of HADS at admission (range 0–21); (7) physical summary score of the Short Form of the Health Status measure, SF-36 [21] (range 0–100); (8) mental summary score of the SF-36 questionnaire (range 0–100); (9) avoidance score of the extended, 22 items, Impact of Event Scale (IES-R) [22, 23] (range 0–40); (10) intrusion score of the IES-R [22, 23] (range 0–40); (11) hyper-arousal score of the IES-R [22, 23] (range 0–30); (12) perceived severity of injury (binary variable: very light to moderate vs. severe to very severe); (13) perceived expected injury outcome (binary: soon recovered or getting better vs no recovery or worsening).

The analyses were adjusted for the following potential confounders, which are likely to be associated with the predictive variables and RTW: gender, age at admission (treated as continuous variable), clinic, native language (local language of the clinic location, i.e. French or German, versus other), marital status (living in stable partnership versus alone), educational level (≤9 years vs. >9 years), time between accident and admission in clinic (<12 months vs. >12 months), possession of a work contract at admission (yes vs. no), trauma localization : upper limb, lower limb, neck, low back (three binary dummy variables, with upper limb as the reference category).

### Statistical Analysis

The associations between the binary outcome variable RTW at 2 years and the exposure variables were evaluated by means of logistic regression. We built statistical models as follows. First, predictors were tested individually, once alone and once adjusted by the confounders. Second, all predictors with *p* ≤ 0.25 in the previous adjusted models were tested together and with the confounders in what we call a full model. Third, in a backward selection procedure, we dropped from the full model the predictor with the highest *p* value. After dropping a predictor, the Akaike information criterion (AIC) was noted. AIC is a measure of how close outcomes predicted by a model are to the true expected outcomes and a lower AIC indicates a better model fit. This procedure was repeated until a group of predictors remained that could not be further reduced without increasing the AIC compared to the full model. At this point only predictors with *p* < 0.1 remained in the model, which we call the minimal model. The previously dropped predictors were then individually added to the minimal model, to make sure that their effect remained statistically not significant (*p* > 0.1) and that their presence did not alter the effects of the minimal predictors of the minimal model, which became final.

The relationships between continuous predictors and confounders and the probability of RTW were found to be acceptably linear after comparing the deviances of models containing the best fractional polynomial transformations of these variables and those of models with untransformed variables [24]. Thus neither transformation nor recoding was needed to achieve linearity. However, continuous predictor variables were z-score transformed, to produce odds-ratios related to comparable increments in different variables. A z-score indicates the deviation from the variable’s mean expressed as number of standard deviations.

The quality of our final model was assessed by the Hosmer–Lemeshow goodness of fit statistic [25] and the concordance index, also called c statistic. The c statistic is a measure of the predictive ability of a prognostic model and is equivalent to the area under the receiver operating characteristics (ROC) curve [25]^.^ In our analyses, the c statistic is the ability of a model to discriminate subjects with high probability of RTW from those with low probability. The c statistic ranges from 0.5 (no predictive discrimination) to 1 (perfect discrimination); values below 0.7 indicate a poor discrimination ability, values 0.7–0.8 an acceptable discrimination, and values above 0.8 an excellent to outstanding discrimination. In general, prognostic models tend to produce higher c statistics in the dataset in which the model was developed than they will in future subjects. Therefore, to assess the internal validation of our final model we computed an optimism-corrected c statistic using bootstrapping [26].

Expecting medical and cultural differences between two clinics located in two different linguistic areas, we considered patients to be correlated within clinic. Therefore, a clustered sandwich estimator was used to estimate the variance–covariance matrix and the coefficients’ standard errors in all regression models. This procedure affects only the standard errors but not the regression coefficients. The option vce (cluster) was used within the logistic command of the statistical package Stata. Stata version 11.2 was used for all calculations (StataCorp, College Station, TX, USA, www.stata.com).

## Results

**Fig. 1:**
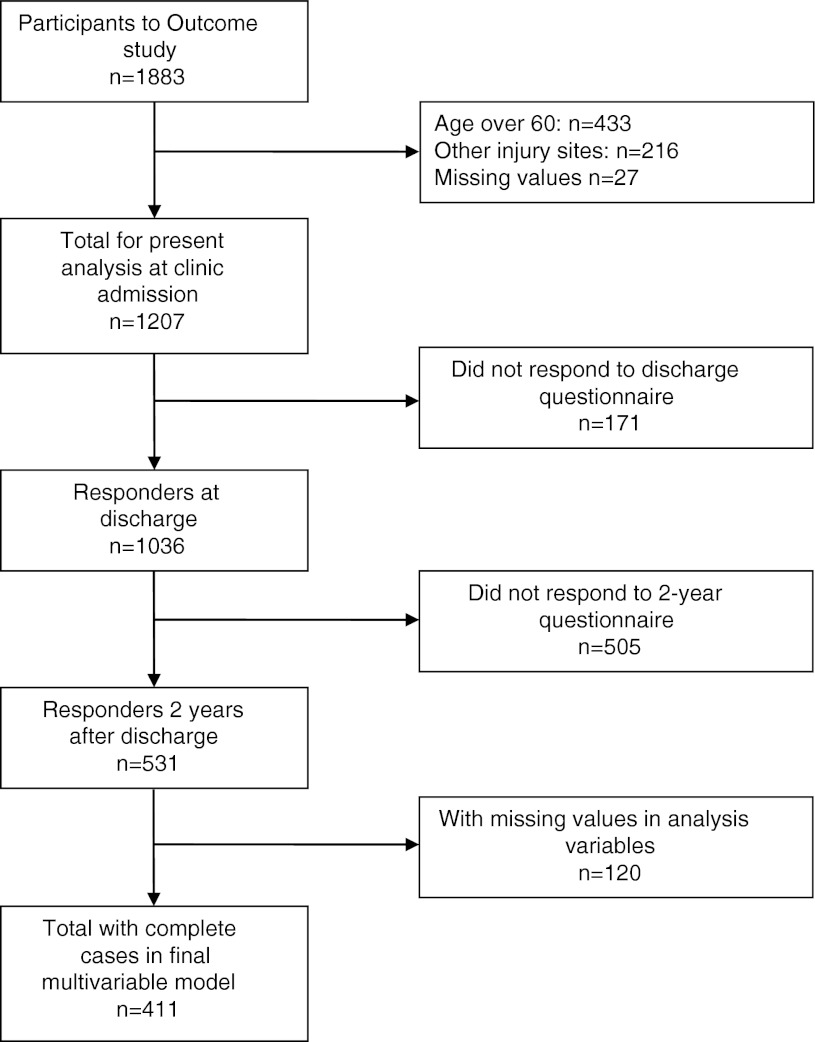
Flow chart

1,883 Patients participated in the Outcome cohort. After excluding subjects older than 60 years or with injury locations other than limbs or back, or with missing values for these variables, 1,207 patients were entered in the present study (Fig. 1). 665 (55 %) were recruited by the RKB and 542 (45 %) by the CRR.

At admission, the mean age of the 1,207 participants was 41 years, SD 11. The proportion of males was 81 %. Moreover, 55 % of the patients spoke the local language (i.e. German or French), 53 % were married or had a domestic partner and 47 % were alone. 45 % patients had more than 9 years education, 64 % were in possession of a work contract. The sites of the injury were: 26 % upper limb, 34 %lower limb, 17 % neck and 22 % low back (Table 1).

**Table 1:** Summary statistics for the confounding variables

Variable	Category	Admission (n = 1,207)		2-year responders (n = 531)		2-year complete cases (n = 411)	
		Frequency	%	Frequency	%	Frequency	%
Clinic	RKB	665	55.1	290	54.6	249	60.6
	CRR	542	44.9	241	45.4	162	39.4
Gender	Men	979	81.1	423	79.7	336	81.7
	Women	228	18.9	108	20.3	75	18.3
Age	NA	Mean = 41.4	SD = 10.6	Mean = 43.5	SD = 10.2	Mean = 43.3	SD = 10.3
Native language	Local	665	55.1	370	69.7	296	75.0
	Other	542	44.9	161	30.3	115	28.0
Marital status	Single	569	47.1	231	43.5	169	41.1
	Married/partnership	638	52.9	300	56.5	242	58.9
Education	≤9 years	594	49.2	249	46.9	203	50.6
	>9 years	544	45.1	253	47.6	208	49.4
	Missing values	69	5.7	29	5.5	NA	NA
Time between accident and admission	<12 months	481	39.8	219	41.2	182	44.3
	>12 months	695	57.4	296	55.7	229	55.7
	Missing values	34	2.8	16	3.0	NA	NA
Work contract at admission	Yes	776	64.3	368	69.3	297	72.3
	No or not known	362	30.0	140	26.4	114	27.7
	Missing values	69	5.7	23	4.3	NA	NA
Main traumatic localization	Upper limb	315	26.0	137	25.8	102	24.8
	Lower limb	416	34.4	206	38.8	158	38.4
	Neck	207	17.1	83	15.6	72	17.5
	Low back	272	22.5	105	19.8	79	19.2

At discharge, 171 patients did not respond to the investigation questionnaire and another 505 did not send back the 2-year questionnaire. Thus, 531 patients responded at 2 years, 411 of whom could be included in the final model having complete data (Fig. 1).

The values of the confounding variables did not change substantially between admission and 2 years post discharge with the exception of native language. The proportion of native speakers was 55 % at admission, 68 % at 2 years, and 75 % of the complete cases (Table 1).

The values of the predictors did not change substantially between the admission set and the final analysis set (Table 2).

**Table 2:** Summary statistics for the predictive variables

Variable	Category	Admission (n = 1,207)			2-year responders (n = 531)			2-year complete cases (n = 411)	
		n^a^	Mean	SD	n^a^	Mean	SD	Mean	SD
General health at admission	NA	1,198	47.8	22.0	526	49.6	22.1	49.1	21.9
General health improvement during stay	NA	1,025	5.4	24.6	517	6.9	23.4	6.7	23.7
Pain at admission	NA	1,174	55.7	25.2	511	52.5	25.9	53.1	25.2
Pain decrease during stay	NA	981	−5.6	22.9	490	−5.6	24.1	−6.0	23.9
HADS anxiety	NA	1,196	9.1	4.5	526	8.6	4.4	8.6	4.4
HADS depression	NA	1,196	7.4	4.5	525	6.9	4.3	7.0	4.3
SF-36 physical summary score	NA	1,151	33.3	7.0	503	33.3	7.1	32.9	6.8
SF-36 mental summary score	NA	1,151	37.8	9.1	503	39.0	9.1	39.1	9.1
IES-R avoidance	NA	1,174	11.5	10.4	517	10.8	10.6	11.1	10.6
IES-R intrusion	NA	1,173	12.5	11.2	517	11.2	10.9	11.6	10.9
IES-R hyper-arousal	NA	1,164	10.8	8.6	513	10.0	8.4	10.4	8.5

			Frequency	%		Frequency	%	Frequency	%
Perceived severity of injury	Severe to very severe	1,207	753	62.2	531	312	58.8	248	60.3
	Very light to moderate		457	37.8		219	41.2	163	39.7
Expected outcome	Deterioration or no improvement	1,207	381	31.5	531	146	27.5	114	27.7
	Soon recovered or getting better		829	68.5		385	72.5	229	72.3

^a^All predictors could not be assessed for all patients due to missing values

### Predictors of Return to Work

**Fig. 2:**
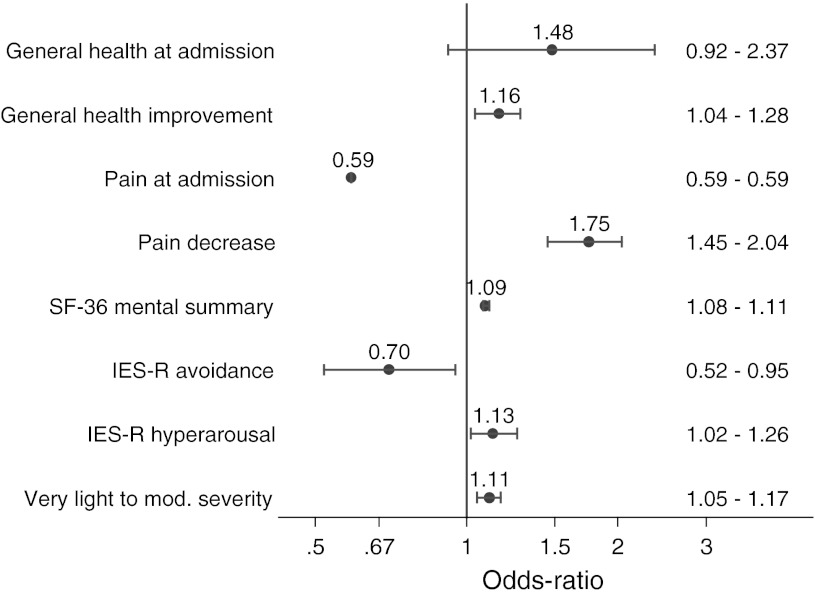
Confounder-adjusted odds-ratios (OR) and 95 % confidence intervals for the predictors of the final, multivariable, model (see “Methods”); OR for continuous variables refer to increments of one SD. A value over the unity indicates an increased likelihood to return to work. X-axis in logarithmic scale. N = 411

After the simple regressions and the regressions with the single predictors adjusted for the confounders, all predictors (except the interval between the accident and the hospitalisation) were used to build the full multiple regression model (Table 3). After the variable selection procedure, a final model containing eight variables was obtained (Fig. 2).

**Table 3:** Logistic regressions

Variable	Category	Non-adjusted (simple regressions)				Fully adjusted (complete model) n = 411		
		n	OR	CI 95 %	P	OR	CI 95 %	P
General health at admission	NA	526	1.66	1.26–2.18	<0.001	1.40	0.89–2.21	0.149
General health improvement during stay	NA	517	1.19	1.02–1.37	0.022	1.14	0.98–1.33	0.082
Pain at admission	NA	511	0.62	0.60–0.64	<0.001	0.61	0.57–0.65	<0.001
Pain decrease during stay	NA	490	1.37	1.35–1.39	<0.001	1.69	1.47–2.04	<0.001
HADS anxiety	NA	526	0.64	0.49–0.83	0.001	0.94	0.86–1.02	0.139
HADS depression	NA	525	0.60	0.51–0.71	<0.001	1.06	0.83–1.36	0.634
SF-36 physical summary score	NA	503	1.48	1.15–1.92	0.002	1.16	0.75–1.76	0.498
SF-36 mental summary score	NA	503	1.41	1.40–1.42	<0.001	1.15	0.96–1.37	0.123
IES-R avoidance	NA	517	0.69	0.51–0.92	0.011	0.69	0.61–0.79	<0.001
IES-R intrusion	NA	517	0.70	0.52–0.94	0.016	1.05	0.60–1.84	0.865
IES-R hyper-arousal	NA	513	0.70	0.55–0.87	0.002	1.32	0.76–1.68	0.535
Perceived severity of injury	Severe or very severe	312	1			1		
	Very light to moderate	219	1.97	1.11–3.49	0.020	1.08	1.03–1.14	0.002
Expected outcome	Deterioration or no improvement	146	1			1		
	Soon recovered or getting better	385	2.41	1.86–3.13	<0.001	1.14	0.78–1.66	0.492

OR for continuous variables refer to one-SD increments

In the fully adjusted model, predictors are adjusted for each other and for the confounding variables (for the final model see Fig. 2)

Among the subjects included in the final model, 238 (58 %) were back to work, while 173 (42 %) were not working. Compared to those not working, the subjects back to work had higher general health score at admission (mean = 52.9, SD = 20.6, vs. mean = 43.9, SD = 22.7), higher general health improvement during stay (mean = 7.9, SD = 21.4, vs. mean = 5.1, SD = 26.5), lower pain score at admission (mean = 48.6, SD = 25.3, vs. mean = 59.4, SD = 23.6), higher pain decrease during stay (mean = −9.1, SD = 23.7, vs. mean = −1.7, SD = 23.7), higher SF-36 mental health score (mean = 40.4, SD = 9.5, vs. mean = 37.4, SD = 8.3), lower IES-R avoidance (mean = 9.1, SD = 9.9, vs. mean = 13.9, SD = 10.9) and IES-R hyperarousal (mean = 8.9, SD = 8.2, vs. mean = 12.4, SD = 8.4) scores and higher proportion perceiving injury severity as very light to moderate (46 vs. 31 %).

After controlling for the confounders, the data were compatible with higher chances of being at work for patients with a higher perceived general health at admission (OR = 1.48 for 1-SD increments), although a lack of effect could not be excluded (see confidence interval). The chances of being at work were also higher for patients whose general health improved during stay (OR = 1.16 for 1-SD increments), those with a higher SF-36 mental summary score (OR = 1.09 for each SD), higher IES-R hyperarousal score (OR = 1.13 for each SD) and those feeling their injury severity as very light to moderate (OR = 1.11 for comparison with severe to very severe) (Fig. 2).

On the contrary, patients with higher pain at admission (OR = 0.59 for each SD), pain increase during stay (OR = 0.57 for each SD) or higher IES-R avoidance score (OR = 0.70 for each SD) had lower chances to be back at work.

The Hosmer–Lemeshow statistic provided no strong evidence that the final model failed at predicting the data (χ^2^ = 393.92, df = 392, *p* = 0.463). The final model’s apparent c statistic was 0.78 while the optimism-corrected c statistic, calculated with 200 bootstrap resamples, was 0.69.

## Discussion

In the present study, we observed that good perception of general health and low pain may predict a higher RTW 2 years after a rehabilitation program. These results confirm those of others for pain [7, 27–29] and self rated health [30, 31]. Moreover, in accordance with earlier results [17, 18] changes in pain and perceived health, during hospitalisation, were also predictors of RTW. It means that, among patients with an identical initial pain, those who experience a larger decrease in pain during hospitalisation are more prone to return to work 2 years later. From these results, it can be hypothesized that an intervention on pain and perceived health may favour return to work but this issue remains to be investigated more thoroughly. A complementary variable to health perception is the perceived severity of the lesion. The present study suggests that patients who believe their lesions are very light to moderate (compared to severe or very severe) have increased likelihood to RTW. These results are compatible with those of others [32].

Among biomedical variables, the demographic factor most commonly found to be associated with chronic disability is older age [33, 34] or age below 41 years (and gender [35]). For this reason, these variables were taken as confounders in the present study. The important fraction of male patients (i.e. 81 %) is expected because these patients are mostly blue collar workers victim of work or traffic accidents. It appears from the literature that pain and psychosocial factors are of primary importance to explain long term sick leave [36–38]. Moreover, general health perception and perception of health change/improvement was strongly associated with a duration of sickness absence and with recurrence of new sick leave episodes for the same musculoskeletal complaints [31, 39]. To our knowledge, this is the first study to identify sub scores of the IES-R questionnaire, an indicator of the post traumatic stress disorder (PTSD) (for a review see [40]) as predictors of RTW in patients with traumatic injuries. The cut off value of 33, identifies the PTSD with a sensitivity of 0.91 and a specificity of 0.82 [41]. Interestingly, our population had a high mean total score of the IES-R (i.e. 35 ± 28 [SD]). More precisely the IES avoidance (e.g. effortful avoidance of situations that are reminders of the accident) and hyperarousal (e.g. being irritable, having trouble falling asleep, watchful and on guard) was retained in our final model. Apparently, our results are not in accordance with those of Toien et al. [16] who reported that IES was not selected in their model prediction of RTW in trauma patients 1 year after the first assessment. However, in the latter study, the total score of the non revised version of the IES was used, which may be less sensitive to identify intrusion and avoidance [42]. Moreover, total IES-R score may not be appropriate in the prediction of the outcome because each sub-score (i.e. avoidance, hyperarousal and intrusion) may behave differently. For instance, from results of the final minimal model in the present study, patients with hyperarousal problems were apparently more prone to RTW (Fig. 2). These results are surprising because the opposite effect can be expected. However, the non-adjusted simple regression presents an odds ratio of 0.7 for the IES-R hyperarousal score, i.e. a lower likelihood of RTW. To explore this point, models were calculated, containing IES-R hyperarousal plus the confounders and all possible combinations of 1–4 of the remaining final-model predictors. The OR for IES-R hyperarousal switched from negative to positive only in the presence of the IES-R avoidance variable. Thus, IES-R avoidance seems to be an important confounder of IES-R hyperarousal. Interestingly, clinical practice confirms the possibility of patients with hyperarousal symptoms to develop strategies compatible with professional environment. This empirical experience of our medical staff, which is used to treat patients with PTSD, is compatible with the concept that some hyperarousal behaviour may not be deleterious for RTW. Moreover, a study performed on Oklahoma city bombing survivors [43] has reported that patients with avoidance behaviour received more mental health treatment, had much more interference with activities and were more dissatisfied with work than those with an hyperarousal behaviour. However, we cannot fully explain our finding and this issue has to be investigated further. Finally, it must be kept in mind that a clinical diagnosis of PTSD cannot be formally determined from the response of the IES-R questionnaire. Consequently, further research should be performed in which trained clinicians may systematically screen this pathology in patients. A 1-year follow-up study performed on patients with back and/or neck pain, found strong associations between pain, expectancy, pain-related fear and a belief in an underlying and serious medical problem [29]. These associations can also be extended to PTSD [44] and are also compatible with the present results. These results suggest that patients’ pain care should also involve the treatment of fear avoidance [45], PTSD and some other psychological aspects.

Our prediction of RTW by the SF-36 mental summary is in accordance with the results by Schultz et al. [46] who identified the same variable as predictor of RTW 3 months after injury and with those of Pattani who observed that poor quality of life (especially the mental health component) at baseline and without improvement, predicts return to work [47].

A strength of the present work is the duration of the observation (2 years) because there seem to be a lack of studies with a long follow-up period (i.e. more than 1 year) [48].

The main limitation of this study is the low response rate of the eligible patients 2 years after hospitalisation i.e. 34 %. In our study, the descriptive statistics of responder, predictors and confounders were relatively stable through time except for the proportion of local native language speakers. It has been reported that patients with local native language were more prone to RTW [49] and also more likely to respond to questionnaires [19]. This suggests that data were not missing at random and, therefore, loss to follow-up caused some degree of bias in our OR estimations. Furthermore, because the RTW was obviously only assessed from the responders, our results probably overestimate overall RTW proportion and means and proportions of predictor values. Another limitation of this protocol is that these results cannot be extended to all patients with musculoskeletal injuries. However, because only patients with persistent health problems after an accident are hospitalised in our clinic, the results can be useful for this kind of patients who are following treatment for long periods.

Unfortunately, for technical reasons the present study did not assess the working status continuously but only at 2 years by sending questionnaires for the following reasons: some patients did not know the exact date of return to work and continuous follow up was a too demanding protocol. Moreover, the files of our insurance company did not allow finding the date of RTW i.e. only rents and work ability were available. Our patients often exhibit trauma associated with psychological, social and occupational problems, Consequently, it is not surprising that an important fraction of those patients did not return to work after 2 years.

In conclusion, this study assessed predictors of RTW on patients with musculoskeletal injuries 2 years after a rehabilitation program. Patients who reported a higher general health perception and a low pain at the start of rehabilitation period as well as those who exhibit a large general health improvement and pain decrease during rehabilitation were more prone to return to work. Conversely, individuals who presented avoidance behaviour had a low probability to return to work. Our findings suggest that rehabilitation interventions should also depend on the patient general health perception as well as pain and fear-avoidance beliefs related to the accident.
